# Gas flow through atomic-scale apertures

**DOI:** 10.1126/sciadv.abc7927

**Published:** 2020-12-18

**Authors:** Jothi Priyanka Thiruraman, Sidra Abbas Dar, Paul Masih Das, Nasim Hassani, Mehdi Neek-Amal, Ashok Keerthi, Marija Drndić, Boya Radha

**Affiliations:** 1 Department of Physics and Astronomy, University of Pennsylvania, Philadelphia, PA 19104, USA.; 2 Department of Electrical and Systems Engineering, University of Pennsylvania, Philadelphia, PA 19104, USA.; 3 Department of Physics and Astronomy, School of Natural Sciences, University of Manchester, Oxford Road, Manchester M13 9PL, UK.; 4 National Graphene Institute, University of Manchester, Manchester M13 9PL, UK.; 5 Department of Basic Sciences and Humanities, University of Engineering and Technology, New Campus, GT Road Lahore, Kala Shah Kaku, Pakistan.; 6 Department of Physics, Shahid Rajaee Teacher Training University, 16875-163 Lavizan, Tehran, Iran.; 7 Universiteit Antwerpen, Groenenborgerlaan 171, B-2020 Antwerpen, Belgium.; 8 Department of Chemistry, School of Natural Sciences, University of Manchester, Oxford Road, Manchester M13 9PL, UK.

## Abstract

Gas flows are often analyzed with the theoretical descriptions formulated over a century ago and constantly challenged by the emerging architectures of narrow channels, slits, and apertures. Here, we report atomic-scale defects in two-dimensional (2D) materials as apertures for gas flows at the ultimate quasi-0D atomic limit. We establish that pristine monolayer tungsten disulfide (WS_2_) membranes act as atomically thin barriers to gas transport. Atomic vacancies from missing tungsten (W) sites are made in freestanding (WS_2_) monolayers by focused ion beam irradiation and characterized using aberration-corrected transmission electron microscopy. WS_2_ monolayers with atomic apertures are mechanically sturdy and showed fast helium flow. We propose a simple yet robust method for confirming the formation of atomic apertures over large areas using gas flows, an essential step for pursuing their prospective applications in various domains including molecular separation, single quantum emitters, sensing and monitoring of gases at ultralow concentrations.

## Teaser

Atomically narrow apertures show fast transport of helium.

## INTRODUCTION

Understanding confined gas flows in angstrom-scale tight spaces plays a major role not only in the design of gas extraction techniques but also for gas separation and production (*1*). In extremely narrow pores, the mean free path of a gas is much larger than that of the dimensions of the pore itself, which leads to gas dynamics dominated by molecular collisions with walls of the pore rather than the intermolecular collisions (*2*). This is known as the free molecular regime, and the gas flux through these pores was comprehensively described using the Knudsen equation (*2*), which has been modified and adapted to explain the flows through various confined systems (*3*). From a theory standpoint, a pore or an aperture is a simple model system through which gas transmission is proportional to the impingement of gas molecules, i.e., likelihood of a gas molecule encountering a pore, and the activation barrier, if any, to cross the pore. In the cases where the membrane surface can adsorb gases, the flow is a combination of direct transmission through the pore and diffusion along the membrane surface (*4*). Despite the emergence of many nanoscale gas flow conduits such as nanopores (*5*–*9*), nanotubes (*10*–*12*), nanochannels (*11*, *13*–*16*), nanolaminates (*17*, *18*), etc., ultimately narrow quasi–zero-dimensional (0D) apertures with atomic-scale dimensions in both the transmembrane and lateral directions, which challenge the applicability of the Knudsen equation for gas flows, have been limited (*5*, *8*, *9*, *19*). Although atomic vacancies in monolayer 2D materials have been ideal candidates for theoretical simulations and modeling of gas flows (*4*, *20*–*22*), they have not been studied extensively in experiments (*9*, *14*). Here, we investigate an inert gas, i.e., helium flow through atomic vacancies in freestanding monolayer tungsten disulfide (WS_2_) membranes, to validate the Knudsen description in the ultimate atomic aperture limit.

## RESULTS

Several studies in the past have explored various sources for atomic defect creation in 2D materials; among those, the popular techniques are oxygen plasma (*23*, *24*), thermal annealing (*25*), ion and electron beam irradiation (*26*, *27*), acid etching (*23*, *28*), and ultraviolet-induced oxidation etching (*5*). In particular, ion irradiation offers a precise method of creating atomic vacancies with a controlled localization of defect sites at comparatively high densities (>10^11^ cm^−2^). Recently, we have illustrated how to create highly controlled single-atom defects by focused ion beam (FIB) irradiation on a monolayer transition metal dichalcogenide (TMD) flake (*29*). In particular, TMDs provide better imaging contrast in aberration-corrected scanning transmission electron microscopy (AC-STEM) imaging and appear less prone to contamination, enabling easier characterization of the defects. We compared the effects of FIB irradiation on various suspended monolayer TMDs and established that defects with areas down to 8 Å^2^ (single transition metal vacancy) can be produced in WS_2_ with a specific low irradiation dose (increasing dose produces larger vacancies in the membrane). Therefore, we choose monolayer WS_2_ as an optimal base support for hosting single-atom defects. Apart from being mechanically stable with a Young’s modulus of 270 GPa and a thickness of ~0.7 nm (*30*), our detailed investigation of the controlled ion irradiation mechanism minimizes the possibility of undesired damage or tears. The FIB irradiation mechanism used here allows for exclusively hosting single atomic apertures on a freestanding monolayer WS_2_ membrane, enabling a high density of uniform defects in the range of 9 × 10^11^ ± 3.5 × 10^10^ cm^−2^ (see sections S1 to S3). These defect densities are comparable to that achieved in graphene membranes (*9*).

**Fig. 1:**
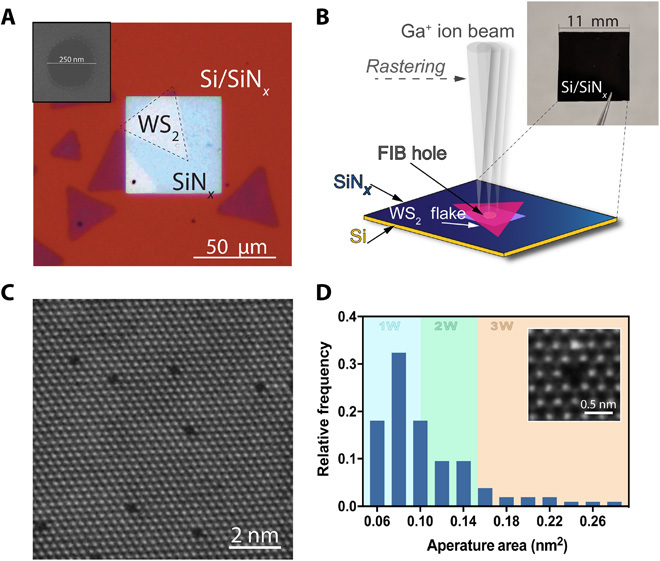
Atomic apertures fabricated using an FIB scanning technique. (**A**) Optical microscope of a monolayer WS_2_ flake suspended in the center of a silicon nitride (SiN*_x_*) membrane (~50-nm thickness, 50 μm by 50 μm) using position-control transfer technique. The inset shows a scanning electron microscopy image of a hole of 250 nm in diameter drilled in the center of the SiN*_x_* membrane. (**B**) Schematics of the irradiation technique on the suspended WS_2_ flake, and the inset shows a photograph of a SiN*_x_*/Si chip (11 mm by 11 mm). (**C**) Aberration-corrected high-angle annular dark-field (AC-HAADF) image of irradiated monolayer WS_2_ flake at a dose of ~5.1 × 10^13^ ions/cm^2^. Bright spots indicate W atoms. (**D**) Histogram of apertures produced with the irradiated dose used in (C). Light shading of blue, green, and orange indicate the size ranges of 1W, 2W, and 3W atomic apertures in the graph, respectively. Inset: A high-magnification AC-HAADF STEM image of one such aperture.

The WS_2_ membranes incorporating atomic vacancies are supported on silicon chips (SiN*_x_*/Si) with a size of 11 mm by 11 mm (Fig. 1A and fig. S1). In the center of each chip, one or more submicrometer holes were drilled in a freestanding silicon nitride (SiN*_x_*) membrane (Fig. 1B). A monolayer WS_2_ flake was suspended on submicrometer holes present on these silicon chips (see fig. S1). The suspended flake was irradiated with a 30-kV gallium–sourced FIB under specific precalibrated irradiation dose conditions to produce single atomic vacancies (illustration is shown in Fig. 1B). We exposed samples to an ion irradiation dose of 5.1 × 10^13^ ion/cm^2^ and obtained a defect density of 0.08 ± 0.03%, with an average defect area of 0.12 nm^2^ and a median defect area of 0.09 nm^2^. Figure 1C is a representative image of atomic-scale defects produced through this method. We observe single atomic apertures where 1W atom vacancies can be seen, as included in Fig. 1D. It is often challenging to locate S atoms in AC-STEM images because of their weaker contrast compared to heavier W atoms. The creation of atomic defects possibly leads to reconstruction of immediate surrounding sulfurs in the monolayer lattice; hence, we see a variety of shapes of the apertures such as triangular, truncated triangular, to pseudo-spherical (see table S1 and fig. S4). On the basis of analysis of several atomic-resolution images, we estimate the total defect sites resulting from missing W atoms to be few hundreds to few thousands per one device depending on the supported membrane area.

**Fig. 2:**
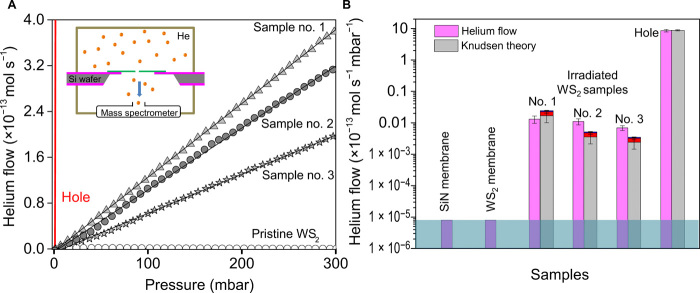
Gas flow through atomic apertures. (**A**) Helium gas permeation measured through atomic apertures in WS_2_ made with same irradiation dose (5.1 × 10^13^ ions/cm^2^) but with different WS_2_ areas leading to difference in the number of defects in a given sample. The proportion of the 1W, 2W, and 3W vacancies in each sample is 69:22:9%, sample no. 1, *N* ~ 2070 ± 830; sample no. 2, *N* ~ 440 ± 170; sample no. 3, *N* ~ 300 ± 120. Pristine WS_2_ membranes without any irradiation and large bare hole not covered with WS_2_ (shown in red color) are shown alongside as controls. Solid lines running through symbols indicate best fit to the data. Inset shows a schematic of our experimental setup. (**B**) Comparison of normalized permeance per unit pressure of irradiated samples and controls, with the Knudsen estimates. The colors within the gray bars representing Knudsen estimates arise from the flow contributions due to 2W (red) and 3W (dark blue) vacancies. Horizontal light blue color bar indicates the detection limit. Error bars on the helium flow of samples are from the SD of flow values recorded over a series of helium flow measurements (measured three times) on the same sample repeated after annealing three times. For the Knudsen theory estimates, the error bars arise from the error values associated with the number of defects and with the total area of the apertures *A* in each sample.

The relatively large number of atomic apertures (up to ~2000) in our samples enables gas flows detectable by conventional mass spectrometers, such as helium leak detector. Our membranes are mechanically robust and sustained the pressure differences of up to ~1 bar. For gas flow measurements, the experimental setup consists of mounting the silicon chips with O-rings to separate two vacuum chambers, one held at variable pressure *P* and the other in high vacuum connected to a mass spectrometer as depicted in the inset of Fig. 2A (also see fig. S5). The samples are well sealed such that atomic apertures in the WS_2_ membrane act as the only connecting paths between the two chambers where gas molecules can flow. As control samples, three replicas were done for each of the pristine silicon nitride membrane with and without holes and nonirradiated WS_2_ membrane suspended over the silicon nitride hole. The control samples used to establish baseline flow are a different set of membranes than those used for gas transport; however, they all come from the same fabrication procedures and chemical vapor deposition (CVD) growth. A bare hole without WS_2_ layer exhibits large gas flow, as expected, and has been used as a standard leak for validation of the experimental setup (Fig. 2). A freestanding pristine WS_2_ monolayer covering nine holes each with diameters of ~250 nm, without any irradiated defects, exhibited negligible helium flow below our detection limit (~10^−18^ mol s^−1^ mbar^−1^). It is remarkable that the WS_2_ monolayer grown by CVD methods (see fig. S2) has such low intrinsic defect density that it is practically impermeable over a suspended area of a few square micrometers. Let us recall the ultralow permeability, ranging from 10^−23^ mol s^−1^ mbar^−1^ to only few gas molecules per hour (*31*, *32*), of intrinsic defects in 2D materials with high crystal quality such as mechanically exfoliated graphene, studied extensively with specialized device architectures and atomic force microscopy measurements done over several days. Apart from the high crystal quality of our WS_2_ monolayer, the impermeability emphasizes the excellent sealing of the WS_2_ layer on the SiN*_x_* membrane in our devices, which is achieved by repeated annealing of the samples in H_2_/Ar atmosphere at 350°C, both right after the WS_2_ monolayer transfer and also before the gas flow measurements (see section S3).

**Fig. 3:**
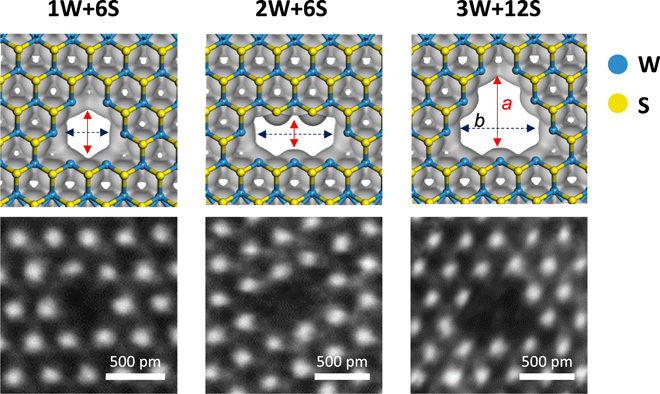
Atomic apertures and electron density isosurfaces. Typical aperture sizes with the irradiation dose (~5.1 × 10^13^ ions/cm^2^) are shown. The top panel shows the density functional theory (DFT) results for electron density isosurfaces of atomic apertures in a WS_2_ membrane (isovalue of 0.2 *e*/Å^3^). The S and W atoms are shown in yellow and blue, respectively. The blue and red arrows inside the images are the accessible dimensions in width *a* and length *b* across the pores (1W+6S, *a* ≈ *b* ~ 3.2 Å; 2W+6S, a ~ 2.1 A, b ~ 6.2 A; 3W+12S, a ~ 5.8 A, b ~ 6.2 A). The accessible aperture areas for helium molecules are ~0.08, ~ 0.13, ~ 0.23 nm^2^, respectively. The bottom row includes the AC-STEM images of the defect, while the top row illustrates the observed atomic configuration.

Next, we did helium (He) gas flow measurements on FIB-irradiated samples containing atomic vacancy defects in suspended WS_2_ membranes. Three typical irradiated samples along with controls are shown in Fig. 2A, in which sample no. 1 has WS_2_ suspended on nine holes each with a diameter of ~200 nm. From our fabrication method, mainly three types of defects are possible such as 1W (~69%), 2W (~22%), and 3W (~9%) defects, respectively. Here, the pore configuration is focused on W atoms since experimentally, the AC-STEM contrast from S atoms is weak, and hence, their locations are not identified with certainty. Unlike the geometric area given in Fig. 1D, to get the effective area (accessible pore area for He atom), the van der Waals (vdW) diameter of each atom on the aperture edge is subtracted (see section S2) (*14*). The total estimated tungsten defects is given as *N* = *N*1*+ N*2*+ N*3, where *N*1, *N*2, and *N*3 are estimated numbers of 1W, 2W, and 3W vacancies, respectively, from AC-STEM image analysis. In our samples, *N* ~ 2070 ± 830 for sample no. 1; the sample no. 2 was WS_2_ on a single hole with a diameter of 250 nm to yield total defects *N* ~ 440 ± 170; the sample no. 3 was WS_2_ on a 200-nm-diameter hole to give estimated defects, *N* ~ 300 ± 120. He flow through atomic apertures increases linearly with increasing pressure and also with increasing number of defects in a sample (Fig. 2A). The gas flux is not exactly linearly correlated with the increase in *N*. The additional sources of error include the variation in the substrate hole area leading to an overall WS_2_ suspended membrane area variation and errors in the ion irradiation dose, in total amounting to about ~40% error. At the experimental working pressure *P* ranges from few to 200 mbar, the mean free path of helium is >0.5 μm, and the Knudsen number for these atomic apertures in our WS_2_ monolayer is >10^3^. Here, the defects are not circular and have a well-defined atomic structure (see inset in Figs. 1D and 3), meaning that a diameter (typically used for larger circular pores) is not an optimal measure of size. We quote their geometric area, i.e., mean area <*A*>, and the characteristic sizes in table S1. As an example, the 1W (+6S) defect has a pore with characteristic size, ~3.15 Å, and an area of 0.08 nm^2^, whereas the 3W (+6S) defect has a pore size of ~5.25 Å and an area of 0.216 nm^2^. To represent the size of the He, we use the kinetic diameter (~2.6 Å), which is a semiclassical notion; however, for monoatomic spherical molecules like He, this is quite close to the quantum-mechanical size of the electron cloud around the nuclei (*33*). As the aperture size is much smaller than the mean free path, the mass flow of the gas *Q* (moles per second) through the aperture is simply the impingement rate upon the area of the pore (see section S6), as described by Knudsen (*22*, *34*).

$$Q=\mathrm{PA}{\left(1/2\pi M\;\mathrm{RT}\right)}^{1/2}$$ (1)

where *P* is the inlet pressure, *A* is the total area of the conducting apertures, *M* is the atomic mass of the gas being transported (*M* = 4 g mol^−1^ for helium), *R* = 8.314 J mol^−1^ K^−1^ is the gas constant, and *T* is the temperature (*T* = 295 K in our experimental setup). In our case, *A* is the sum of all individual atomic aperture areas in the WS_2_ membrane, which is, on average, *N*1 × *A*1 *+ N*2 × *A*2 *+ N*3 × *A*3 (in our samples, *N* can be varied from 300 to 2000 for an individual sample by increasing the membrane area; *A*1, *A*2, and *A*3 are the accessible aperture areas for 1W, 2W, and 3W vacancies, i.e., ~0.08, ~0.13, and ~0.23 nm^2^, respectively, given in table S1. In the case of control devices, i.e., large bare holes, *A* is the sum of individual hole areas (with diameters in the range of ~200 to ~300 nm). Taking into consideration the proportion of the various defects observed in AC-STEM images, we calculated the Knudsen estimates from all the vacancies such that 3W vacancies contribute to ~9% of the flux, whereas ~91% of the flux is contributed by 1W (+6S) and 2W vacancies together (Fig. 2B). If the pores are much narrower than the size of the molecule, then there can be a finite energy barrier, which is expressed as an exponential term in the Eq. 1, exp(−*E*/*RT*), where *E* is the energy barrier that substantially reduces the transmission coefficient of the gas even for small *E* (*14*, *22*).

The 2D membranes with atomic apertures show measurable helium flux ~10^−16^ to 10^−15^ mol s^−1^ mbar^−1^, over two to three orders higher than the detection limit and significantly lower than the leakage rate for submicrometer holes (Fig. 2B). This corresponds to a flow of about ~10^7^ to 10^8^ He molecules per second per millibar pressure difference across the membrane. Theoretical simulations with such small pores in graphene have estimated a pass through frequency of 10^4^ He molecules per second per defect (close to our flow values per millibar for a sample with ~2000 defects; Fig. 2), which can be likely ascribed to a low diffusion barrier for He resulting from its noninteracting nature (*14*, *20*). As a check for the reproducibility of devices with atomic apertures, we would like to point out that in a given batch of irradiated samples, about 40 to 50% samples show the measurable flow commensurate with the number of defects estimated by the irradiation dose (*29*). Other devices either did not conduct gas or showed much higher gas flow. From the He flow, we back-calculated the number of defects contributing to the flow using Eq. 1, and *N* matches close to the defect numbers from AC-STEM image analysis for the sample nos. 1 to 3. For high leaking samples, substituting experimental *Q* in Eq. 1 gave large contributing defect area *A*, which does not correlate with the defect density statistics attained from AC-STEM, hinting that these samples might have tears or cracks in the membrane. Examination of these samples by scanning electron microscopy (SEM) on large areas indeed indicates that they are damaged samples (see tears in suspended WS_2_; fig. S6).

The measured He flow values are within an order of magnitude of the values predicted by the Knudsen estimates, despite the uncertainties in our defect densities and the distribution of the type of defects. Equation 1 is based on the kinetic theory and has been applied to describe the flows through apertures using effusion mechanism in both theoretical (*4*) and experimental literature (*5*, *6*). To more precisely validate the agreement to Eq. 1 and/or evaluate its limitations in our experiments, we would need to further decrease our experimental sources of error, including the calibration of all defect areas, which is a challenging experimental task. To verify whether there is any energy barrier, we measured gas flows by varying the temperature between ~25° to ~100°C, by using a heating tape wound around the chamber, interfaced with a temperature controller. We did not see noticeable variation in the gas flow with temperature, hinting an absence of an energy barrier (an exponential dependence is expected if an energy barrier is present). As per Eq. 1, a *T*^−1/2^ dependence would lead to variation of flux by <10% within the temperature range tested, and this is beyond our experimental measurement error.

From the electron density isosurface (EDI) of defects shown in Fig. 3 (and fig. S4) using first-principles density functional theory (DFT) calculations, accessible pore areas are obtained (table S1). The accessible pore areas from EDI closely match with those estimated using vdW radii (section S2). The surface transport contributions can be negligible as He does not adsorb sufficiently on the WS_2_ basal plane, similar to that observed on graphene (typical adsorption layer thickness, ~4 to 6 Å) (*35*).

Ideal atomic apertures with only 1W missing and with no missing sulfurs on the pore edge would likely, theoretically, be nonconducting. On the other hand, 1W defects with six sulfur vacancies with an accessible pore diameter of ~3.2 Å, which is larger than the kinetic diameter of helium (2.6 Å), would be amenable to gas flows. As shown in Fig. 1D, the minimum area of the defects obtained from AC-STEM imaging was about 0.06 to 0.1 nm^2^, which corresponds to the defects with 1W and 6S missing (~0.08 nm^2^), whereas the 1W defects with no sulfurs missing would be of significantly lower area (~0.002 nm^2^). As the characteristic pore size decreases, the electron overlap between He and membrane significantly increases, thereby increasing the energy barriers for the flow. However, the theoretical calculation of energy barriers might not necessarily capture all the favorable electronic interactions in real systems as there can be rearrangements in the vacancies. These discrepancies between the computed energy barriers and the experimental flows have been noted before by Wang *et al* (*8*). Specifically, our results do not imply a significant energy barrier compared to theoretical predictions (*4*, *8*, *14*, *22*), and we also note that different levels of DFT calculations (and supercell size, or equivalently, the ratio between the pore area and the simulated defect area in membrane) can give different energy barriers; thus, a separate systematic DFT study is required for carefully probing the energy barriers.

We stored our samples with atomic vacancy defects under ambient conditions. When we tested the samples after a few weeks, it was observed that they tend to get clogged with time, leading to reduced/no He flow. As is well known, all surfaces are likely to be covered by hydrocarbons, which might cause the clogging (*15*) of atomic apertures in our case. However, upon reannealing at 350°C, He flow was regained to the previously observed value within a factor of 2. Storage of samples in activated charcoal helped extend the life span of the atomic apertures to be open for few months. Annealing with a combination of storage in charcoal has enabled us to keep the atomic apertures open for about a year. The atomic vacancy defects, once created, are quite stable and show similar helium flow with repeated annealing, indicating that the atomic apertures do not expand or propagate rapidly. Those samples that have been irradiated but did not conduct gas remained nonconductive even after repetitive annealing. We speculate that these samples could have contained a majority of the dominant 1W defect type with one “W” atom missing but with partial or all S atoms at that site, thereby increasing the energy barrier and leading to no He flux. While the ion irradiation dose is optimized for single-atom (W) defect pores, the removal of sulfur atoms from ion irradiation and the subsequent AC-STEM imaging to precisely know the composition of sulfur vacancies at a defect site have remained a challenge. However, in the extreme limit, when there were large holes, tears, or cracks in the membrane, the flow was much higher, and the membranes themselves were not stable. In addition, we performed ion flow measurements in KCl solution on our atomic apertures as a cross-check. Those samples that showed inconsistent and unexpectedly high gas flows showed high ionic conductance (~400 nS in 1 M KCl; see fig. S7), again indicating the presence of tears (visible in SEM; see fig. S6). The samples that showed gas flows commensurate with the Knudsen equation exhibited only a small ionic conductance, <1 nS in 0.1 M KCl for total membrane area ~0.4 to 0.6 mm^2^, as shown in fig. S7. This is expected as the atomic apertures with sizes <6 Å exclude ions (*27*), but the presence of tears in the membranes leads to bulk ionic flow (see fig. S6). These observations further emphasize that the sealing of WS_2_ monolayers to the SiN*_x_* membranes is leak proof, leaving no gaps, and hence, only irradiated defects contribute to the observed gas flow.

## DISCUSSION

Let us analyze the gas flow in nano- and angstrom-scale pores from the literature, in comparison to our atomic vacancy defects presented here. It has been shown in the literature that when the pores are less than the size of the gas molecule passing through (*8*), activated transport is observed and highly sensitive energy barriers play a major role in the transport, and the barrier is usually estimated using combined theoretical and experimental efforts. In this work, since the atomic vacancy apertures are only slightly larger than the gas molecule (i.e., less than twice the size of helium molecule, 2.6 Å), the flow is governed by simple effusion (*14*). In general, effusive flow is seen as detrimental and the cause of reduction for selectivity between gases in size exclusion. Because of this, an accurate comparison of the observed permeability and value estimated from the Knudsen description for the known size of the aperture is often overlooked. Such a comparison made for nanometer-sized holes showed the validity of the Knudsen description in graphene pores down to ~7 nm in size, where a *N*_2_ permeance of ~0.05 mol m^−2^ s^−1^ Pa^−1^ was observed (*6*). Angstrom-size defects (size, ~0.38 to ~0.43 nm) made by ozone-induced etching in a CVD-grown graphene layer were shown to have a H_2_ permeance of about 10^−7^ mol m^−2^ s^−1^ Pa^−1^ (*9*), which is only one order higher than the permeability of graphene membrane, 10^−8^ mol m^−2^ s^−1^ Pa^−1^ hosting the defects (*36*). However, the observed gas flow values for atomic defects are still much lower than that estimated from the Knudsen description for given defect densities, which might be due to the overestimation of the conducting apertures. In our WS_2_ atomic aperture samples, the He gas flow obtained matches the Knudsen estimates within a factor of 2 to 3, and the gas flow normalized by the total area of defects translates to ~0.05 to 0.1 mol m^−2^ s^−1^ Pa^−1^. From theoretical studies on graphene pores with sizes 3.6 to 4.8 Å, for defect densities up to ~10^14^ cm^−2^, large permeance of ~0.1 mol m^−2^ s^−1^ Pa^−1^ has been predicted (*14*, *20*), which closely matches with our experimental values. These permeance values are higher than those typically observed in silica (*37*), zeolite, and metal-organic framework membranes, graphene oxide membranes (*18*), which are in the range of ~10^−6^ to 10^−7^ mol m^−2^ s^−1^ Pa^−1^. Large permeance values are obtained through our WS_2_ apertures, even when normalized with the area of the membrane, i.e., ~10^−4^ to 10^−5^ mol m^−2^ s^−1^ Pa^−1^, which indicates that most of the atomic apertures are in the predicted size range and hence conductive to gases.

In conclusion, we demonstrate the fast He transport through quasi-0D atomic-scale apertures (~W sites). To our knowledge, this is the first experimental observation on He gas transport in such angstrom-scale pores in WS_2_/TMD monolayers where we attempt to evaluate the applicability of the Knudsen equation down to the ultimate limit. Our results indicate the necessity of future theoretical models to explore the role of sensitive variation of the energy barriers of various gas molecules and their critical dependence on the size of the aperture at this atomic limit, especially taking the electron density isosurfaces of atoms to account for the enhanced flux. In addition, this work provides a new method for corroborating atomic pore formation and their density over large areas via a simple bulk measurement technique of measuring gas flow through them. This is analogous to using nanoholes (few hundred nanometers in size) as standard leak elements for calibration (*38*). So far, the only way to inspect and confirm the atomic pore formation in the case of atomic vacancy defects is by AC-STEM, which is limited to relatively small areas. Conventional ionic transport measurements through nanopores are mediated by Hille’s equation (*27*), which yields a linear relationship between conductance and total aperture area. However, for pores that are smaller in dimensions than many common salts, ionic conductance measurements do not yield a predictable or a measurable signal, leading to the breakdown of Hille’s model for atomic aperture limit (*28*). With our study, we would like to highlight that once the pore creation is confirmed and calibrated by AC-STEM locally, gas flow measurements can act as a standard to test for the presence of these pores and their density over large areas. Let us also note that the stability of pore/aperture configuration of these atomic scale defects needs further investigation (*27*, *39*) as the pores could change over time, which can be attempted in future experiments by imaging the pores over time. More advanced methods including dynamic scanning tunneling microscopy need to be devised to locate S atoms (*40*) and also any other atoms such as possible contaminants (C, O, and N) to be certain of pore compositions and sizes. Future work may involve efficient gas separation investigations using a scaled-up version of such membranes.

## MATERIALS AND METHODS

### Sample fabrication

#### *CVD growth of monolayer WS*_2_ *flakes*

Monolayer WS_2_ flakes were grown using CVD processes similar to previously reported methods (*27*, *29*). Solutions of 2% sodium cholate growth promoter and 15 × 10^−3^ M ammonium heptamolybdate (metatungstate) were spun onto piranha-cleaned silicon (Si) substrates coated with 150 nm of SiO_2_, which were then loaded into the center of a tube furnace (Thermo Fisher Scientific Lindberg/Blue M). For WS_2_, samples were heated in argon atmosphere [100 standard cubic centimeters per minute (sccm)] at a rate of 65°C min^−1^ and held at 800°C for 10 min, during which time 15 sccm of H_2_ was also added. Approximately 100 mg of sulfur precursor placed 22 cm from the substrates was kept at 180°C during the growth procedures. Both samples were rapidly cooled to room temperature by cracking open the furnace.

#### *Position-controlled transfer of WS*_2_ *flakes*

With WS_2_ flakes grown on Si/SiO_2_ wafer pieces, we cut smaller pieces with areas about 3 × 3 mm^2^ that contain good WS_2_ flakes. Using a wet transfer technique, the TMD flake side of the SiO_2_ pieces is coated with poly(methyl methacrylate) (PMMA) (MicroChem C4, 4000 rpm for 60 s). The PMMA-coated substrates are left to dry at ambient temperature for a few minutes before being floated onto the KOH etchant (8 g per 100 ml of water). Depending on the thickness of the SiO_2_ wafer, the time for etching away the SiO_2_ layer varies. Last, we observe the PMMA + TMD flake floating on the KOH etchant. These PMMA + TMD flakes are then rinsed three times in clean water baths before being used to transfer them onto a SiN*_x_*/Si chip with a hole (diameter range, 200 to 300 nm) drilled by FIB. Position transfer is practiced when the flake size and their number density are high, so that in this case, it is possible to place one PMMA + TMD flake onto the SiN*_x_* FIB hole. This step of placing the PMMA + TMD flake over the SiN*_x_* FIB hole is somewhat challenging since the coverage of the FIB hole depends on the number of the as-grown flakes. However, because in a given batch of CVD growth, the number of grown flakes is large, the transfer is completed within several transfer trials. In addition to this manual transfer, we have also used custom-built manipulators, with needles and tweezers to move the flake on the substrate to increase device yield. These manipulations came at a cost, since there was now a higher risk of device failure caused by the usage of additional tools, which can sometimes break the membrane. In this work, all devices were fabricated by manually “fishing” (moving) the PMMA + TMD flake onto the SiN*_x_* FIB hole. After we transfer the flake and optically verify the coverage, the sample is then dried in ambient temperature for 30 min. Furthermore, the sample is placed into hot acetone (90°C) to remove the supporting PMMA.

#### Gallium ion irradiation

Monolayer TMD flakes were irradiated with a Ga^+^-sourced ion beam FEI Strata Dual-Beam instrument. The acceleration voltage of the ion beam was set to 30 kV and incident normal to the surface. The beam spot size was observed to be 100 nm for a flash second at 10-pA current. To produce atomic defects, an area of 250 nm by 250 nm was irradiated with the dwell time (16 μs) and current (10 pA). Pixel resolution (1024 × 884) was kept constant. The exposure was carried out in an imaging mode, which followed a raster pattern where the beam sequentially exposed each pixel in a row. The instrument, FEI FIB Strata DB 235, has an option to “grab frame,” which takes a single scan at a set resolution; this option was used for all the scans. The dose was varied by changing the number of scans. Suspended and substrate-supported samples were exposed to FIB irradiation while sitting on holey carbon TEM grids and Si/SiO_2_ substrates, respectively.

#### AC-STEM imaging

AC-STEM images of WS_2_ samples were acquired using a Cs-corrected JEOL ARM 200CF STEM operating at 80 kV. Images were obtained using a high-angle annular dark-field (HAADF) detector with a collection angle of 54 to 220 mrad and 10-cm camera length. Probe current (22 pA), focusing time (<2 s), and electron dose (≈6.0 × 10^6^ *e*^−^ nm^−2^) were kept low to minimize beam-induced knock-on damage.

### First-principles calculations

The DFT was performed with the generalized gradient approximations (*41*) form with exchange-correlation potential parametrization of Perdew-Burke-Ernzerhof (*42*). The calculations were carried out by the Quantum ESPRESSO package (*43*) and have been performed on the basis of the plane-wave basis sets and ultrasoft nonlocal pseudopotentials (*44*). To take the vdW contributions into account in the total energy, the Tkatchenko-Scheffler (*45*) method was used. The cutoff of kinetic energy in the plane-wave expansion and the convergence threshold for the self-consistent field calculations were chosen as 280.0 and 10^−6^ eV per atom, respectively. The WS_2_ membrane was relaxed both in plane and out of plane.

Calculations were done at 0 K for a rectangular supercell shape with size 15.953 × 16.579 Å^2^ for all considered defects consisting of 29(60), 29(54), 28(58), 28(54), 27(54), and 27(48) atoms of W (S) for 1W, 1W (+6S), 2W (+2S), 2W (+6S), 3W (+6S), and 3W (+12S) with a vacuum layer of 30 Å between periodic images in the vertical direction. We calculated the EDI using the above aforementioned functionals and energy cutoff. For the EDI images (Fig. 3 and fig. S4), the isovalue was set to be 0.2 *e*/Å^3^.

## SUPPLEMENTARY MATERIALS

Supplementary material for this article is available at http://advances.sciencemag.org/cgi/content/full/6/51/eabc7927/DC1

## References

[1] H. B. Park, J. Kamcev, L. M. Robeson, M. Elimelech, B. D. Freeman, Maximizing the right stuff: The trade-off between membrane permeability and selectivity. Science 356, eaab0530 (2017). 28619885 10.1126/science.aab0530

[2] M. Knudsen, Die Gesetze der Molekularströmung und der inneren Reibungsströmung der Gase durch Röhren. Ann. Phys. 333, 75–130 (1909).

[3] M. V. Smoluchowski, Zur kinetischen Theorie der Transpiration und Diffusion verdünnter Gase. Ann. Phys. 338, 1559–1570 (1910).

[4] C. Sun, M. S. H. Boutilier, H. Au, P. Poesio, B. Bai, R. Karnik, N. G. Hadjiconstantinou, Mechanisms of molecular permeation through nanoporous graphene membranes. Langmuir 30, 675–682 (2014). 24364726 10.1021/la403969g

[5] S. P. Koenig, L. Wang, J. Pellegrino, J. S. Bunch, Selective molecular sieving through porous graphene. Nat. Nanotechnol. 7, 728–732 (2012). 23042491 10.1038/nnano.2012.162

[6] K. Celebi, J. Buchheim, R. M. Wyss, A. Droudian, P. Gasser, I. Shorubalko, J.-I. Kye, C. Lee, H. G. Park, Ultimate permeation across atomically thin porous graphene. Science 344, 289–292 (2014). 24744372 10.1126/science.1249097

[7] Y. Yamada, K. Murota, R. Fujita, J. Kim, A. Watanabe, M. Nakamura, S. Sato, K. Hata, P. Ercius, J. Ciston, C. Y. Song, K. Kim, W. Regan, W. Gannett, A. Zettl, Subnanometer vacancy defects introduced on graphene by oxygen gas. J. Amer. Chem. Soc. 136, 2232–2235 (2014). 24460150 10.1021/ja4117268

[8] L. Wang, L. W. Drahushuk, L. Cantley, S. P. Koenig, X. Liu, J. Pellegrino, M. S. Strano, J. Scott Bunch, Molecular valves for controlling gas phase transport made from discrete ångström-sized pores in graphene. Nat. Nanotechnol. 10, 785–790 (2015). 26237344 10.1038/nnano.2015.158

[9] J. Zhao, G. He, S. Huang, L. F. Villalobos, M. Dakhchoune, H. Bassas, K. V. Agrawal, Etching gas-sieving nanopores in single-layer graphene with an angstrom precision for high-performance gas mixture separation. Sci. Adv. 5, eaav1851 (2019). 30746475 10.1126/sciadv.aav1851 PMC6357726

[10] B. J. Hinds, N. Chopra, T. Rantell, R. Andrews, V. Gavalas, L. G. Bachas, Aligned multiwalled carbon nanotube membranes. Science 303, 62–65 (2004). 14645855 10.1126/science.1092048

[11] J. K. Holt, H. G. Park, Y. Wang, M. Stadermann, A. B. Artyukhin, C. P. Grigoropoulos, A. Noy, O. Bakajin, Fast mass transport through sub-2-nanometer carbon nanotubes. Science 312, 1034–1037 (2006). 16709781 10.1126/science.1126298

[12] L. Ge, A. Du, M. Hou, V. Rudolph, Z. Zhu, Enhanced hydrogen separation by vertically-aligned carbon nanotube membranes with zeolite imidazolate frameworks as a selective layer. RSC Adv. 2, 11793–11800 (2012).

[13] R. W. Baker, *Membrane Technology and Applications* (John Wiley & Sons, Ltd., ed. 2, 2004).

[14] L. Wang, M. S. H. Boutilier, P. R. Kidambi, D. Jang, N. G. Hadjiconstantinou, R. Karnik, Fundamental transport mechanisms, fabrication and potential applications of nanoporous atomically thin membranes. Nat. Nanotechnol. 12, 509–522 (2017). 28584292 10.1038/nnano.2017.72

[15] A. Keerthi, A. K. Geim, A. Janardanan, A. P. Rooney, A. Esfandiar, S. Hu, S. A. Dar, I. V. Grigorieva, S. J. Haigh, F. C. Wang, B. Radha, Ballistic molecular transport through two-dimensional channels. Nature 558, 420–424 (2018). 29925968 10.1038/s41586-018-0203-2

[16] G. Scorrano, G. Bruno, N. di Trani, M. Ferrari, A. Pimpinelli, A. Grattoni, Gas flow at the ultra-nanoscale: Universal predictive model and validation in nanochannels of ångstrom-level resolution. ACS Appl. Mater. Interfaces 10, 32233–32238 (2018). 30185043 10.1021/acsami.8b11455 PMC6836450

[17] H. W. Kim, H. W. Yoon, S.-M. Yoon, B. M. Yoo, B. K. Ahn, Y. H. Cho, H. J. Shin, H. Yang, U. Paik, S. Kwon, J.-Y. Choi, H. B. Park, Selective gas transport through few-layered graphene and graphene oxide membranes. Science 342, 91–95 (2013). 24092738 10.1126/science.1236098

[18] H. Li, Z. Song, X. Zhang, Y. Huang, S. Li, Y. Mao, H. J. Ploehn, Y. Bao, M. Yu, Ultrathin, molecular-sieving graphene oxide membranes for selective hydrogen separation. Science 342, 95–98 (2013). 24092739 10.1126/science.1236686

[19] M. H. Khan, M. Moradi, M. Dakhchoune, M. Rezaei, S. Huang, J. Zhao, K. V. Agrawal, Hydrogen sieving from intrinsic defects of benzene-derived single-layer graphene. Carbon 153, 458–466 (2019).

[20] S. Blankenburg, M. Bieri, R. Fasel, K. Müllen, C. A. Pignedoli, D. Passerone, Porous graphene as an atmospheric nanofilter. Small 6, 2266–2271 (2010). 20814926 10.1002/smll.201001126

[21] D.-e. Jiang, V. R. Cooper, S. Dai, Porous graphene as the ultimate membrane for gas separation. Nano Lett. 9, 4019–4024 (2009). 19995080 10.1021/nl9021946

[22] L. W. Drahushuk, M. S. Strano, Mechanisms of gas permeation through single layer graphene membranes. Langmuir 28, 16671–16678 (2012). 23101879 10.1021/la303468r

[23] S. C. O’Hern, M. S. H. Boutilier, J.-C. Idrobo, Y. Song, J. Kong, T. Laoui, M. Atieh, R. Karnik, Selective ionic transport through tunable subnanometer pores in single-layer graphene membranes. Nano Lett. 14, 1234–1241 (2014). 24490698 10.1021/nl404118f

[24] J. Jadwiszczak, C. O’Callaghan, Y. Zhou, D. S. Fox, E. Weitz, D. Keane, C. P. Cullen, I. O’Reilly, C. Downing, A. Shmeliov, P. Maguire, J. J. Gough, C. McGuinness, M. S. Ferreira, A. L. Bradley, J. J. Boland, G. S. Duesberg, V. Nicolosi, H. Zhang, Oxide-mediated recovery of field-effect mobility in plasma-treated MoS_2_. Sci. Adv. 4, eaao5031 (2018). 29511736 10.1126/sciadv.aao5031 PMC5837433

[25] J.-A. Ke, S. Garaj, S. Gradečak, Nanopores in 2D MoS_2_: Defect-mediated formation and density modulation. ACS Appl. Mater. Interfaces 11, 26228–26234 (2019). 31305058 10.1021/acsami.9b03531

[26] Z. Bai, L. Zhang, H. Li, L. Liu, Nanopore creation in graphene by ion beam irradiation: Geometry, quality, and efficiency. ACS Appl. Mater. Interfaces 8, 24803–24809 (2016). 27572502 10.1021/acsami.6b06220

[27] J. P. Thiruraman, K. Fujisawa, G. Danda, P. M. Das, T. Zhang, A. Bolotsky, N. Perea-López, A. Nicolaï, P. Senet, M. Terrones, M. Drndić, Angstrom-size defect creation and ionic transport through pores in single-layer MoS_2_. Nano Lett. 18, 1651–1659 (2018). 29464959 10.1021/acs.nanolett.7b04526

[28] P. Masih Das, J. P. Thiruraman, Y.-C. Chou, G. Danda, M. Drndić, Centimeter-scale nanoporous 2D membranes and ion transport: Porous MoS_2_ monolayers in a few-layer matrix. Nano Lett. 19, 392–399 (2019). 30532980 10.1021/acs.nanolett.8b04155

[29] J. P. Thiruraman, P. Masih Das, M. Drndić, Irradiation of transition metal dichalcogenides using a focused ion beam: Controlled single-atom defect creation. Adv. Func. Mater. 29, 1904668 (2019).

[30] K. Liu, Q. Yan, M. Chen, W. Fan, Y. Sun, J. Suh, D. Fu, S. Lee, J. Zhou, S. Tongay, J. Ji, J. B. Neaton, J. Wu, Elastic properties of chemical-vapor-deposited monolayer MoS_2_, WS_2_, and their bilayer heterostructures. Nano Lett. 14, 5097–5103 (2014). 25120033 10.1021/nl501793a

[31] J. S. Bunch, S. S. Verbridge, J. S. Alden, A. M. van der Zande, J. M. Parpia, H. G. Craighead, P. L. McEuen, Impermeable atomic membranes from graphene sheets. Nano Lett. 8, 2458–2462 (2008). 18630972 10.1021/nl801457b

[32] P. Z. Sun, Q. Yang, W. J. Kuang, Y. V. Stebunov, W. Q. Xiong, J. Yu, R. R. Nair, M. I. Katsnelson, S. J. Yuan, I. V. Grigorieva, M. Lozada-Hidalgo, F. C. Wang, A. K. Geim, Limits on gas impermeability of graphene. Nature 579, 229–232 (2020). 32161387 10.1038/s41586-020-2070-x

[33] N. Mehio, S. Dai, D.-e. Jiang, Quantum mechanical basis for kinetic diameters of small gaseous molecules. J. Phys. Chem. A 118, 1150–1154 (2014). 24446751 10.1021/jp412588f

[34] M. Knudsen, Die Molekularströmung der Gase durch Offnungen und die Effusion. Ann. Phys. 333, 999–1016 (1909).

[35] Y. Tao, Q. Xue, Z. Liu, M. Shan, C. Ling, T. Wu, X. Li, Tunable hydrogen separation in porous graphene membrane: First-principle and molecular dynamic simulation. ACS Appl. Mater. Interfaces 6, 8048–8058 (2014). 24621326 10.1021/am4058887

[36] S. Huang, M. Dakhchoune, W. Luo, E. Oveisi, G. He, M. Rezaei, J. Zhao, D. T. L. Alexander, A. Züttel, M. S. Strano, K. V. Agrawal, Single-layer graphene membranes by crack-free transfer for gas mixture separation. Nat. Commun. 9, 2632 (2018). 29980683 10.1038/s41467-018-04904-3 PMC6035196

[37] R. M. de Vos, H. Verweij, High-selectivity, high-flux silica membranes for gas separation. Science 279, 1710–1711 (1998). 9497287 10.1126/science.279.5357.1710

[38] V. Ierardi, U. Becker, S. Pantazis, G. Firpo, U. Valbusa, K. Jousten, Nano-holes as standard leak elements. Measurement 58, 335–341 (2014).

[39] S. Wang, H. Li, H. Sawada, C. S. Allen, A. I. Kirkland, J. C. Grossman, J. H. Warner, Atomic structure and formation mechanism of sub-nanometer pores in 2D monolayer MoS_2_. Nanoscale 9, 6417–6426 (2017). 28463370 10.1039/c7nr01127j

[40] Y. Wen, C. Ophus, C. S. Allen, S. Fang, J. Chen, E. Kaxiras, A. I. Kirkland, J. H. Warner, Simultaneous identification of low and high atomic number atoms in monolayer 2D materials using 4D scanning transmission electron microscopy. Nano Lett. 19, 6482–6491 (2019). 31430158 10.1021/acs.nanolett.9b02717

[41] J. P. Perdew, J. A. Chevary, S. H. Vosko, K. A. Jackson, M. R. Pederson, D. J. Singh, C. Fiolhais, Atoms, molecules, solids, and surfaces: Applications of the generalized gradient approximation for exchange and correlation. Phys. Rev. B 46, 6671–6687 (1992). 10.1103/physrevb.46.6671 10002368

[42] D. Rao, R. Lu, Z. Meng, Y. Wang, Z. Lu, Y. Liu, X. Chen, E. Kan, C. Xiao, K. Deng, H. Wu, Electronic properties and hydrogen storage application of designed porous nanotubes from a polyphenylene network. Int. J. Hydrogen Energy 39, 18966–18975 (2014).

[43] P. Giannozzi, S. Baroni, N. Bonini, M. Calandra, R. Car, C. Cavazzoni, D. Ceresoli, G. L. Chiarotti, M. Cococcioni, I. Dabo, A. Dal Corso, S. de Gironcoli, S. Fabris, G. Fratesi, R. Gebauer, U. Gerstmann, C. Gougoussis, A. Kokalj, M. Lazzeri, L. Martin-Samos, N. Marzari, F. Mauri, R. Mazzarello, S. Paolini, A. Pasquarello, L. Paulatto, C. Sbraccia, S. Scandolo, G. Sclauzero, A. P. Seitsonen, A. Smogunov, P. Umari, R. M. Wentzcovitch, QUANTUM ESPRESSO: A modular and open-source software project for quantum simulations of materials. J. Phys. Condens. Matter 21, 395502 (2009). 21832390 10.1088/0953-8984/21/39/395502

[44] D. Vanderbilt, Soft self-consistent pseudopotentials in a generalized eigenvalue formalism. Phys. Rev. B 41, 7892–7895 (1990). 10.1103/physrevb.41.7892 9993096

[45] A. Tkatchenko, M. Scheffler, Accurate molecular van der Waals interactions from ground-state electron density and free-atom reference data. Phys. Rev. Lett. 102, 073005 (2009). 19257665 10.1103/PhysRevLett.102.073005

[46] P. K. Chow, R. B. Jacobs-Gedrim, J. Gao, T.-M. Lu, B. Yu, H. Terrones, N. Koratkar, Defect-induced photoluminescence in monolayer semiconducting transition metal dichalcogenides. ACS Nano 9, 1520–1527 (2015). 25603228 10.1021/nn5073495

[47] A. Beskok, G. E. Karniadakis, W. Trimmer, Rarefaction and compressibility effects in gas microflows. J. Fluids Eng. 118, 448–456 (1996).

[48] T. Wu, D. Zhang, Impact of adsorption on gas transport in nanopores. Sci. Rep. 6, 23629 (2016). 27020130 10.1038/srep23629 PMC4810319

[49] K. Wu, Z. Chen, X. Li, Real gas transport through nanopores of varying cross-section type and shape in shale gas reservoirs. Chem. Eng. J. 281, 813–825 (2015).

